# Cultural safety in radiation therapy education: Identifying knowledge deficits to improve Indigenous health practice

**DOI:** 10.1002/jmrs.819

**Published:** 2024-09-08

**Authors:** Thashmira Naidoo, Crispen Chamunyonga, Julie Burbery, Peta Rutledge

**Affiliations:** ^1^ Faculty of Health, School of Clinical Sciences Queensland University of Technology Brisbane Queensland Australia; ^2^ Radiation Oncology Princess Alexandra Hospital Raymond Terrace South Brisbane Queensland Australia; ^3^ Centre for Biomedical Technologies Queensland University of Technology Brisbane Queensland Australia

## Abstract

Reducing health disparities for Aboriginal and Torres Strait Islander peoples requires the integration of cultural safety into healthcare education. This commentary paper addresses cultural safety in the context of the radiation therapy profession and emphasises the importance of making practitioners aware of the knowledge gaps in healthcare practice. The educational strategies to improve cultural awareness amongst undergraduate students and qualified radiation therapists (RTs) are explored. The authors propose a range of recommendations to enhance cultural awareness amongst RTs in the context of Indigenous Australian care, aimed at promoting improved experiences for Aboriginal and Torres Strait Islander peoples receiving cancer care. Curriculum integration and development of initiatives such as workshops and interactive yarning groups are highlighted as pivotal platforms that foster continuous learning in radiation therapy.

## Graphical abstract

This commentary paper addresses cultural safety in the context of the radiation therapy profession and emphasises the importance of highlighting the impact of knowledge and practice gaps. The educational strategies to improve cultural awareness among undergraduate students and qualified Radiation Therapists (RTs) are explored. Curriculum integration and development of initiatives such as workshops and interactive yarning groups are highlighted as pivotal platforms that foster continuous learning in radiation therapy.

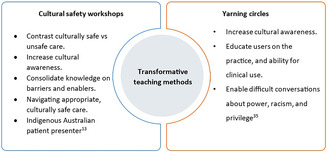

## Introduction

Australia's healthcare system is well‐regarded globally for being accessible, innovative and providing high‐quality service. However, a cultural safety deficit across all areas of health remains a barrier to seeking healthcare interventions.^1^, ^2^, ^3^ For example, Aboriginal and Torres Strait Islander (First Nations) people make up 3.2% of Australia's population and the literature reports that this population faces a disease burden 2.3 times the rate of their counterparts.^4^ In a country as diverse as Australia, health practitioners need to recognise that each patient is unique. This is a crucial first step in creating effective care strategies.

Radiation therapists (RTs) play a significant role amongst the diverse group of allied health practitioners contributing to Australia's healthcare system. As integral members of the healthcare workforce, RTs have a responsibility to ensure that patient‐centric and culturally responsive care is delivered. Through daily interactions with patients, often over a course of several weeks, RTs establish a strong rapport and gain insights into the individual needs of each patient, thus ensuring comprehensive support is provided throughout their treatment journey. To ensure a culturally safe experience is provided to patients, RTs must possess the requisite knowledge and expertise to facilitate effective communication with the patient. This entails understanding and respecting the patient's cultural context, whilst also addressing their specific needs in a compassionate and informed manner. This aligns with the Australian Health Practitioner Regulation Agency (AHPRA) objective of enhancing the Australian health workforce's capacity to deliver culturally safe health services to Aboriginal and Torres Strait Islander peoples.^5^

In the context of Aboriginal and Torres Strait Islander peoples care, there is no research assessing practice and knowledge gaps within the radiation therapy healthcare cohort. However, there is evidence in the literature that highlights knowledge and practice discrepancies within other healthcare professions such as nursing, midwifery, dentistry and others.^6^, ^7^, ^8^, ^9^, ^10^, ^11^, ^12^, ^13^, ^14^ This was found to contribute to a lack of confidence and a sense of inadequacy when delivering patient care from students and practitioners.^7^, ^13^, ^15^, ^16^, ^17^ It is imperative that RTs possess a thorough understanding of First Nations peoples' history and culture to honour delivering culturally safe practice. RT's may undertake trauma informed training to enhance their ability to deliver care is culturally safe and responsive to First Nations patients' needs.^18^

This commentary paper addresses the need for individual RT practitioners to recognise the importance of cultural safety in practice. The authors reviewed the Medical Radiation Practice Board of Australia's (MRPBA) professional capabilities and the literature to highlight the key knowledge gaps identified in the healthcare literature. The authors also discuss the need for educational strategies that target the improvement of cultural awareness in radiation therapy, specifically in the provision of care for First Nations patients.

## Cultural safety in healthcare

Radiation therapists (RTs) are registered professionals governed by National Law under AHPRA working together with the MRPBA to ensure adequacy of training for registered practitioners. The MRPBA defines cultural safety as ‘the ongoing critical reflection of health practitioner knowledge, skills, attitudes, practising behaviours and power differentials in delivering safe, accessible and responsive healthcare free of racism.’^7^ This definition emphasises the necessity for practitioners to recognise and appreciate the distinctions between the practitioner's own culture and that of the patient.^15^, ^18^, ^19^, ^20^, ^21^, ^22^

The MRPBA professional capabilities also address the need for registered practitioners and students to have ‘working knowledge of factors that contribute to and influence the health and wellbeing of Aboriginal and Torres Strait Islander Peoples, including history, spirituality and relationship to land and other determinants of health’.^7^ Cultural safety is discussed under Domain 2 (Professional and Ethical Practitioner) and Domain 3 (Communicator and Collaborator) of the Professional Capabilities. These domains highlight that ‘recognising and evaluating sociocultural factors influencing patient attitude, applying principles of cultural competence and culturally safe care’ are important parts of RT practice, hence the need to ensure that practitioners can a*djust their communication style*.^7^ The emphasis on the need for RTs to possess a comprehensive understanding of factors that influence how patient‐centric care is provided in clinical settings is evident from these definitions.

According to the Australian Institute of Health and Welfare (AIHW), cultural safety is about demonstrating respect for Indigenous cultural values, strengths and diversities.^4^ The AIHW further addresses issues of racism and inequality whilst diminishing power imbalances between practitioners and patients, thereby fostering interactions where authority is shared, and the healthcare system effectively caters to their needs.^4^, ^23^ This perspective also underscores the significance of providing patient‐centric care, a concept that mandates practitioners to comprehend the cultural background and values of the patient.^24^

## Knowledge gaps amongst healthcare practitioners

**Figure 1:**
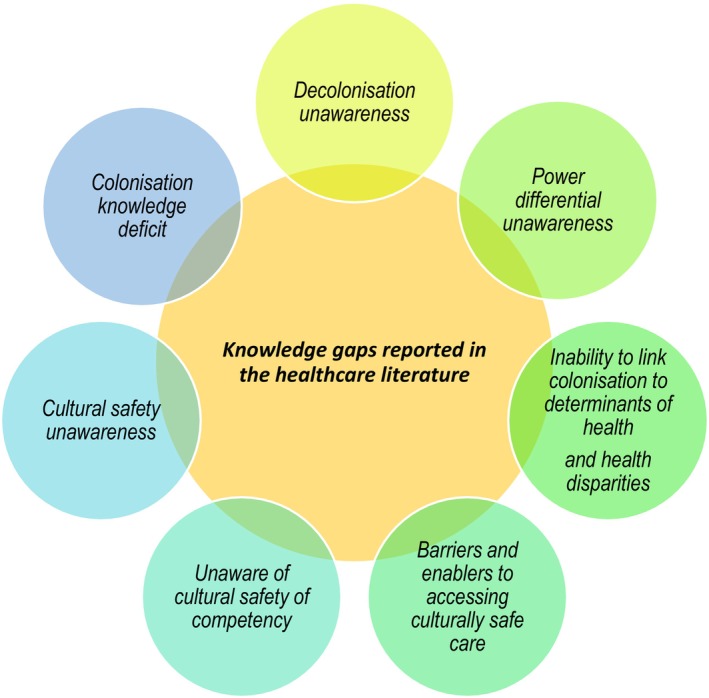
Knowledge gaps observed in qualified healthcare professionals and students which could impact clinical practice.

RTs can draw insights from various healthcare professions which recognise the existing gaps in the knowledge and application of practitioners concerning culturally safe practices when providing care for Aboriginal and Torres Strait Islander communities, due to the lack of RT‐specific literature in this space. In a recent scoping review, Naidoo et al.^25^ commented on the knowledge gaps identified amongst healthcare practitioners and students with respect to Indigenous health. These gaps summarised in Figure 1 were found to impact practising confidence and hence the quality of care.^6^, ^7^, ^9^, ^10^, ^11^, ^12^, ^13^, ^26^When health practitioners are confident in practice, this has been translated to the quality of patient care received and is seen to instil confidence into their patients, improve patient satisfaction and treatment compliance.^9^, ^12^, ^21^, ^27^, ^28^

As highlighted in Figure 1, the knowledge gaps include inadequate understanding of the magnitude of colonisation and its lasting impact on the historical determinants of health, racist systems and laws and ongoing disparities.^6^, ^18^, ^29^ Therefore, as health practitioners we also need to recognise that many Aboriginal and Torres Strait Islander peoples are still dealing with the impacts of intergenerational trauma and ongoing racism. Hence, it is important to acknowledge the enduring effects of intergenerational trauma and persistent racism experienced by many Aboriginal and Torres Strait Islander peoples. All patients rightfully expect their healthcare providers to possess the necessary knowledge and sensitivity to deliver appropriate and responsive patient care.^11^, ^29^

## Educational strategies

The significance of the knowledge deficit observed in the literature supports and warrants the need for a defined level of Indigenous Australian knowledge, required to truly be culturally proficient. The National Framework for Aboriginal and Torres Strait Islander health curriculum^2^ advocates for undergraduate health degrees to focus on developing cultural capability. It is also necessary to produce desired graduate attributes to improve health services delivered to Aboriginal and Torres Strait Island Peoples.^30^, ^31^, ^32^

Gerrad et al.^6^ suggest primarily theory‐based learning as competence‐based cultural development that deflects considerations of bias, privilege and power. In undergraduate settings, teaching and learning can encompass both didactic components and application‐based learning, providing students with opportunities to reflect on patient experiences. However, getting students to have a deep understanding of cultural capability, cultural safety and engage in reflective practice is imperative to minimise implicit generalisations, bias and stereotyping when learning about another culture.^6^

The literature highlights an important tertiary educational deficit amongst undergraduate healthcare students. There is a lack of application‐based learning where fundamental knowledge is confidently translated into practice.^19^ For undergraduate students, clinical placements can be the right environment for the consolidation of knowledge and skill application. Unfortunately, students and experienced health professionals have no guarantee of clinical exposure to all patient demographics.^14^, ^33^, ^34^, ^35^

With limited opportunities to learn and encounter patients during clinical placements, another approach could be to embed cultural safety workshops in university settings. Many studies^12^, ^13^, ^19^ report on the effectiveness of cultural safety workshops. In many instances, the impact of intervention was quantified by utilising pre‐ and post‐assessments. It was found that attendees had an increased awareness and ability to establish an intrinsic link between colonisation and current health inequities by the end of the educational sessions.

In developing educational strategies for qualified RTs, it is also important to learn from other healthcare professions since there are no studies specifically addressing the educational needs of RTs. For example, a study by Durey et al.^13^ highlights how dedicated Indigenous Australian health workshops were implemented for radiation oncology health professionals. They found that most participants significantly benefited from attending a single session, which improved their knowledge, understanding and confidence in distinguishing culturally safe and unsafe practices.^13^ At baseline, less than 10% of participants (*n* = 39) were extremely confident about any one of the assessed 14 items. Post‐workshop, more than 75% of participants were fairly/extremely confident in 11/14 items.^13^

**Figure 2:**
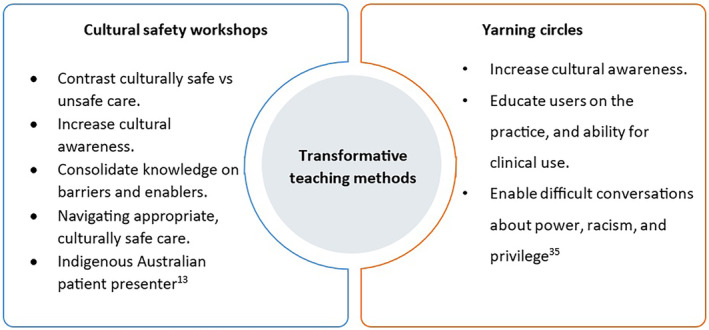
Examples of transformative teaching strategies employed in healthcare to address cultural safety.

Bullen and Robert's study,^10^ employed transformative teaching methods such as intensive workshops and yarning circles. These were consolidated with mixed‐mode theory in which learners benefited from a more immersive, practical cultural capability learning experience.^10^, ^36^ The conducted yarning circles from the literature have been led by an Indigenous Australian educator, an Elder or nominated community member, and in some instances an Indigenous Australian patient who has volunteered their time.^20^, ^28^, ^35^ Figure 2 illustrates how workshops and yarning circles are currently used as educational strategies.

## Barriers to cultural safety education

Despite the required cultural safety and competency requirements stipulated in our professional practice capabilities, it is challenging to define the level or extent of Indigenous health and cultural safety knowledge for registration or practising requirements. The recommendation to define a level of ‘foundational knowledge’ necessary to obtain professional registration comes from the observations of significant knowledge deficits in healthcare students and practitioners regarding Indigenous Australian history.^2^, ^3^, ^10^, ^12^, ^14^, ^18^, ^31^, ^32^, ^37^

In recent years, there has been ongoing debate regarding the shift from cultural competence, as ‘competence’ alludes to a skill that can be fullyattained rather than being understood as a journey of continuous learning.^38^ The differences in adaptations and definitions across regulatory and educational organisations present a challenge, underscoring the imperative of developing a consensus definition for practitioners and educators to refer to. AHPRA's National Scheme's Aboriginal and Torres Strait Islander Health and Cultural Safety Strategy 2020–2025 has identified the necessity to review and establish a widely accepted terminology for cultural safety and cultural competence at a national level. A significant concern highlighted as a ‘medium‐term priority’ for 2018–2019 was the distinction between cultural safety and cultural capability and competence. However, the current MRPBA professional capabilities still encompass the concept of cultural competence.

## Summary and recommendations

In the context of caring for Aboriginal and Torres Strait Islander peoples, it is important to recognise the significance of RTs' awareness of cultural safety. When addressing the gaps in knowledge within education, we must consider avenues for enhancing learning experiences for both undergraduate students and experienced RTs. Developing a culturally competent curriculum that integrates Indigenous perspectives, beliefs and practices into radiation therapy education is necessary. Cultural safety workshops and yarning circles could be used to provide an understanding of Indigenous health issues and cultural safety practices. Extending these initiatives to qualified RTs can significantly contribute to their ongoing education in the format of continuous professional development. Additionally, there is a need for research in radiation therapy to assess practice and knowledge gaps specifically related to the care of Aboriginal and Torres Strait Islander peoples. Such research will help identify specific areas where education and training can be improved and provide evidence‐based recommendations to enhance culturally safe practices within radiation therapy.

## Conclusion

To enhance culturally safe care in radiation therapy, it is crucial to acknowledge the complexities and historical contexts that shape the landscape of healthcare for Aboriginal and Torres Strait Islander peoples. A commitment to continual learning is necessary for RTs to progressively contribute to the advancement of culturally safe practices, ultimately improving the well‐being of Aboriginal and Torres Strait Islander peoples. Addressing the knowledge gaps in the context of radiation therapy necessitates a comprehensive strategy that encompasses both undergraduate students and qualified RTs.

## Conflict of interest

The authors declare no conflict of interest.

## Data Availability

Data sharing not applicable to this article as no datasets were generated or analysed during the current study.

## References

[1] Australian Human Rights Commission . Close the gap: Indigenous health campaign. Australian Human Rights Commission March 18, 2021 [Accessed 2022 January 10]. Available from: https://humanrights.gov.au/our-work/aboriginal-and-torres-strait-islander-socialjustice/projects/close-gap-indigenous-health.

[2] Department of Health . Aboriginal and Torres Strait Islander Health Curriculum Framework. 2014. August 3, 2021 [Accessed 2021 November 12]. Available from: https://www.health.gov.au/sites/default/files/documents/2020/12/aboriginal-and-torres-strait-islander-health-curriculum-framework.pdf.

[3] Davy C , Harfield S , McArthur A , Munn Z , Brown A . Access to primary health care services for Indigenous peoples: A framework synthesis. Int J Equity Health 2016; 15(1): 163. 27716235 10.1186/s12939-016-0450-5 PMC5045584

[4] Australian Institute of Health and Welfare . Culturally safe health care for Indigenous Australians – Australian Institute of Health and Welfare. Australian Institute of Health and Welfare July 7, 2023 [Accessed 2023 July 10]. Available from: https://www.aihw.gov.au/reports/australias-health/culturally-safe-healthcare-indigenous-australians.

[5] Australian Health Practitioner Regulation Agency . The National Scheme's Aboriginal and Torres Strait Islander and Cultural Safety Strategy 2020–2025. December 27, 2020 [Accessed 2021 November 12].

[6] Gerrard JM , Godwin S , Chuter V , Munteanu SE , West M , Hawke F . Release of the National Scheme's Aboriginal and Torres Strait Islander Health and Cultural Safety Strategy 2020–2025; the impacts for podiatry in Australia: A commentary. J Foot Ankle Res 2021; 14: 38. 33971934 10.1186/s13047-021-00466-8 PMC8108329

[7] Medical Radiation Practice Board of Australia . Professional capabilities for medical radiation practitioners. October 7, 2022 [Accessed 2022 November 12]. Available from: https://www.medicalradiationpracticeboard.gov.au/Registration-Standards/Professional-Capabilities.aspx.

[8] Mills K , Creedy DK , Sunderland N , Allen J . Examining the transformative potential of emotion in education: A new measure of nursing and midwifery students' emotional learning in first peoples' cultural safety. Nurse Educ Today 2021; 100: 104854. 33713988 10.1016/j.nedt.2021.104854

[9] Reath J , Abbott P , Kurti L , et al. Supporting Aboriginal and Torres Strait islander cultural educators and cultural mentors in Australian general practice education. BMC Med Educ 2018; 18(1): 236. 30309368 10.1186/s12909-018-1340-x PMC6182837

[10] Bullen J , Roberts L . Transformative learning: A precursor to preparing health science students to work in indigenous health settings? Aust J Indig Educ 2019; 48(2): 129–140.

[11] Tujague NA , Ryan KL . Ticking the box of 'cultural safety' is not enough: Why trauma‐informed practice is critical to Indigenous healing. Rural Remote Health 2021; 21(3): 6411. 34237994 10.22605/RRH6411

[12] Thackrah RD , Wood J , Thompson SC . Cultural respect in midwifery service provision for Aboriginal women: Longitudinal follow‐up reveals the enduring legacy of targeted program initiatives. Int J Equity Health 2020; 19(1): 210. 33238984 10.1186/s12939-020-01325-x PMC7689980

[13] Durey A , Halkett G , Berg M , Lester L , Kickett M . Does one workshop on respecting cultural differences increase health professionals’ confidence to improve the care of Australian Aboriginal patients with cancer? An evaluation. BMC Health Serv Res 2017; 17(1): 660. 28915810 10.1186/s12913-017-2599-z PMC5603013

[14] Mariño R , Satur J , Tuncer E , et al. Cultural competence of Australian dental students. BMC Med Educ 2021; 21(1): 155. 33711993 10.1186/s12909-021-02589-9 PMC7953755

[15] Huria T , Palmer S , Beckert L , Lacey C , Pitama S . Indigenous health: Designing a clinical orientation program valued by learners. BMC Med Educ 2017; 17(1): 180. 28982353 10.1186/s12909-017-1019-8 PMC5629767

[16] Nash D , O'Rourke T , Memmott P , Haynes M . Indigenous preferences for inpatient rooms in Australian hospitals: A mixed‐methods study in cross‐cultural design. HERD 2020; 14(1): 174–189. 32462919 10.1177/1937586720925552

[17] Curtis E , Jones R , Tipene‐Leach D , et al. Why cultural safety rather than cultural competency is required to achieve health equity: A literature review and recommended definition. Int J Equity Health 2019; 18(1): 174. 31727076 10.1186/s12939-019-1082-3 PMC6857221

[18] Yeung S , Bombay A , Walker C , et al. Predictors of medical student interest in Indigenous health learning and clinical practice: A Canadian case study. BMC Med Educ 2018; 18(1): 307. 30547790 10.1186/s12909-018-1401-1 PMC6295008

[19] Withall L , Ryder C , Mackean T , et al. Assessing cultural safety in Aboriginal and Torres Strait Islander Health. Aust J Rural Health 2021; 29: 201–210. 33793013 10.1111/ajr.12708

[20] Ivers R , Jackson B , Levett T , Wallace K , Winch S . Home to health care to hospital: Evaluation of a cancer care team based in Australian Aboriginal primary care. Aust J Rural Health 2019; 27: 88–92. 30694000 10.1111/ajr.12484

[21] Fernando T , Bennett B . Creating a culturally safe space when teaching Aboriginal content in social work: A scoping review. Aust Soc Work 2018; 72(1): 47–61.

[22] McDermott D . “Big Sister” Wisdom: How might non‐Indigenous speech‐language pathologists genuinely, and effectively, engage with Indigenous Australia? Int J Speech Lang Pathol 2019; 21(3): 252–262. 31181968 10.1080/17549507.2019.1617896

[23] Muise GM . Enabling cultural safety in Indigenous primary healthcare. Healthc Manage Forum 2018; 32(1): 25–31. 30304957 10.1177/0840470418794204

[24] Brumpton K , Ward R , Evans R , et al. Assessing cultural safety in general practice consultations for Indigenous patients: Protocol for a mixed methods sequential embedded design study. BMC Med Educ 2023; 23(1): 306. 37131207 10.1186/s12909-023-04249-6 PMC10152729

[25] Naidoo T , Chamunyonga C , Burbery J , Rutledge P . Identifying methods to best integrate indigenous knowledge and perspectives within the radiation therapy undergraduate curriculum. J Med Radiat Sci 2023; 70: 183–191. 36781205 10.1002/jmrs.660 PMC10258646

[26] Zubrzycki J , Shipp R , Jones V . Knowing, being, and doing: Aboriginal and Non‐Aboriginal Collaboration in Cancer Services. Qual Health Res 2017; 27(9): 1316–1329. 28682709 10.1177/1049732316686750 PMC5502907

[27] Mills K , Creedy DK , West R . Experiences and outcomes of health professional students undertaking education on Indigenous health: A systematic integrative literature review. Nurse Educ Today 2018; 69: 149–158. 30081248 10.1016/j.nedt.2018.07.014

[28] Power T , Virdun C , Gorman E , et al. Ensuring Indigenous cultural respect in Australian undergraduate nursing students. High Educ Res Dev 2018; 37(4): 837–851.

[29] Taylor E , Haigh M , Shahid S , Garvey G , Cunningham J , Thompson S . Cancer services and their initiatives to improve the care of indigenous Australians. Int J Environ Res Public Health 2018; 15(4): 717. 29641441 10.3390/ijerph15040717 PMC5923759

[30] West R , Mills K , Rowland D , Creedy DK . Validation of the first peoples cultural capability measurement tool with undergraduate health students: A descriptive cohort study. Nurse Educ Today 2018; 64: 166–171. 29499573 10.1016/j.nedt.2018.02.022

[31] West M , Sadler S , Hawke F , Munteanu SE , Chuter V . Effect of a culturally safe student placement on students' understanding of, and confidence with, providing culturally safe podiatry care. J Foot Ankle Res 2021; 14(1): 9. 33499892 10.1186/s13047-021-00450-2 PMC7836510

[32] Jeffreys M . Teaching cultural competence in nursing and health care, 3rd edn. Springer Publishing Company, New York, 2015.

[33] West R , Wrigley S , Mills K , Taylor K , Rowland D , Creedy DK . Development of a First Peoples‐led cultural capability measurement tool: A pilot study with midwifery students. Women Birth 2017; 30(3): 236–244. 28188040 10.1016/j.wombi.2017.01.004

[34] Mills K , Creedy DK , Sunderland N , Allen J , Carter A , Corporal S . Evaluation of a First Peoples‐led, emotion‐based pedagogical intervention to promote cultural safety in undergraduate non‐Indigenous health professional students. Nurse Educ Today 2022; 109: 105219. 34799192 10.1016/j.nedt.2021.105219

[35] Fleming T , Creedy DK , West R . The influence of yarning circles: A cultural safety professional development program for midwives. Women Birth 2020; 33(2): 175–185. 31053464 10.1016/j.wombi.2019.03.016

[36] Wilson C , Heinrich L , Heidari P , Adams K . Action research to implement an Indigenous health curriculum framework. Nurse Educ Today 2020; 91: 104464. 32526618 10.1016/j.nedt.2020.104464

[37] Fowler AC , Ewens B , Vafeas C , et al. Closing the gap: A whole of school approach to Aboriginal and Torres Strait Islander inclusivity in higher education. Nurse Educ Pract 2018; 30: 86–90. 29660564 10.1016/j.nepr.2018.04.001

[38] Henderson S , Horne M , Hills R , Kendall E . Cultural competence in healthcare in the community: A concept analysis. Health Soc Care Community 2018; 26(4): 590–603. 29516554 10.1111/hsc.12556

